# Enhanced Performance in Fluorene-Free Organometal Halide Perovskite Light-Emitting Diodes using Tunable, Low Electron Affinity Oxide Electron Injectors

**DOI:** 10.1002/adma.201405044

**Published:** 2015-01-09

**Authors:** Robert L Z Hoye, Matthew R Chua, Kevin P Musselman, Guangru Li, May-Ling Lai, Zhi-Kuang Tan, Neil C Greenham, Judith L MacManus-Driscoll, Richard H Friend, Dan Credgington

**Affiliations:** Department of Materials Science & Metallurgy, University of Cambridge27 Charles Babbage Road, Cambridge, CB3 0FS, UK; Department of Physics, University of CambridgeJJ Thomson Avenue, Cambridge, CB3 0HE, UK

**Keywords:** electron injection barrier tuning, perovskite light-emitting diodes, polymer light-emitting diodes, spatial atmospheric atomic layer deposition, zinc magnesium oxide

Organic light-emitting diodes (LEDs) are a multibillion dollar industry, with applications in displays, lighting, and consumer devices.[1] The current limitation is the difficulty in depositing organic emitters by vacuum sublimation over a large area cost effectively.[1]–[3] Solution-processable materials, such as organometal halide perovskites[4] and conjugated polymers[3] have the potential to overcome this limitation, due to their compatibility with roll-to-roll solution-processing techniques and inkjet printing.[2,3,5,6] The development of complementary new electrode materials is essential to increase the performance and commercial potential of LEDs based on these emitters.[3,7]

Organometal halide perovskites used in solar cells have demonstrated an astonishing increase in power conversion efficiency from 4% in 2009 to 19.3% in 2014,[8] while their high photoluminescence quantum efficiency has allowed the first demonstrations of optically pumped lasing.[9,10] More recently, the demonstration of room temperature electroluminescence from this material also promises a bright future for perovskite LED (PeLED) technology.[4] The appealing properties of organometal halide perovskites enabling these advances are their sharp band edges,[11] low non-radiative recombination rates, and suppressed defect formation.[12] In addition, the perovskite band gap can be tuned through its chemical composition, enabling PeLEDs to achieve emission from the infrared to green.[4] Previously, poly(9,9-dioctylfluorene) or F8 was used as a large bandgap charge-blocking layer in PeLEDs, which helps to prevent electrical shorts through the discontinuous perovskite layer, and acts as a spacer to the metal cathode to prevent emission quenching.[4,13] However, F8 is known to be unstable under applied bias in air[14] and its mobility decreases at low temperatures,[15] making it incompatible with the cryogenic analysis techniques typically used to study crystalline semiconductors.[16] Critically, visible-light PeLEDs with F8 show parasitic blue emission due to the formation of excitons in the polyfluorene across the perovskite-free voids, which reduces their color purity.[4] Replacing this organic spacer is therefore the crucial next step in PeLED design. More generally, most perovskite LEDs and solar cells rely on expensive, unstable, and low-conductivity fluorenes for charge injection or extraction, such as 2,2′,7,7′-tetrakis(*N*,*N*-di-*p*-methoxyphenylamine)-9,9′-spiro-bifluorene (spiro-OMeTAD)[17] or F8. Finding new electrode materials that overcome these limitations is thus essential for the commercial application of perovskite optoelectronics.

Stable metal oxide electrodes, such as ZnO, are promising replacements for fluorenes, but are limited by a fixed electron injection level.[3,18] In addition, spray pyrolysis, which is the common method for producing metal oxides in hybrid LEDs, requires deposition temperatures >350 °C. This renders it incompatible with flexible polymer substrates or direct deposition onto perovskites.[3,18,19] In this work, we overcame these limitations by using spatial atmospheric atomic layer-deposited (SAALD) ZnO films, formed at only 60 °C and deposited directly onto green-emitting methylammonium lead tri-bromide (CH_3_NH_3_PbBr_3_) perovskite. Through this, we produced the first PeLEDs not reliant on fluorene-based layers and which were also brighter than the previous report of devices using F8. We found that our new structure presented several advantages. The electron injection barrier with the perovskite could be reduced by incorporating Mg into ZnO to produce Zn_1−*x*_Mg*_x_*O, which decreased the electron affinity from −3.6 to −3.35 eV relative to the vacuum level and reduced the PeLED turn-on voltage. These PeLEDs were stable under bias, and exhibited an electroluminescence peak half as wide as that of conventional InGaN LEDs. The emission was purely from the perovskite (and not contaminated by any emission from the electron injector), which makes PeLEDs with the perovskite/Zn_1−*x*_Mg*_x_*O junction very promising for display applications, as well as electrically pumped lasing. To show that our tunable metal oxide is high quality and can be used in efficient LEDs, we also applied this electron injector to mature polymer LED (PLED) technology, demonstrating luminous efficiencies comparable to or exceeding previous records with hybrid green F8BT and blue aryl-F8:0.5 wt% TFB devices, as well as the ability to significantly reduce the operating voltage by reducing the electron injection barrier.

SAALD ZnO was deposited onto the perovskite using diethylzinc and water vapor precursors to produce a layered structure PeLED: ITO/PEDOT:PSS/CH_3_NH_3_PbBr_3_/ZnO/Ca/Ag (**Figure 1**a), and this successfully led to devices displaying room temperature electroluminescence (Figure 1b). Lowering the deposition temperature from 150 °C to 60 °C significantly improved the luminance from 0.2 to 550 cd m^−2^, whilst maintaining a low turn-on voltage of 2 V (Figure 1b). This performance improvement was due to a reduction in the pore size in the perovskite as the deposition temperature was reduced (Figure 1c–e) and is a notable improvement over the previous report of green PeLEDs using F8, which had a maximum luminance of 364 cd m^−2^.[4] We also note that the PeLEDs with SAALD ZnO deposited on the perovskite had significantly higher performance than PeLEDs with sol-gel TiO*_x_* deposited on the perovskite using a previously reported method,[20] as shown in Figure 1b and Figure S1 (Supporting Information). This was due to solvents from the TiO*_x_* precursor damaging the perovskite layer during deposition (Figure S2, Supporting Information).

**Figure 1:**
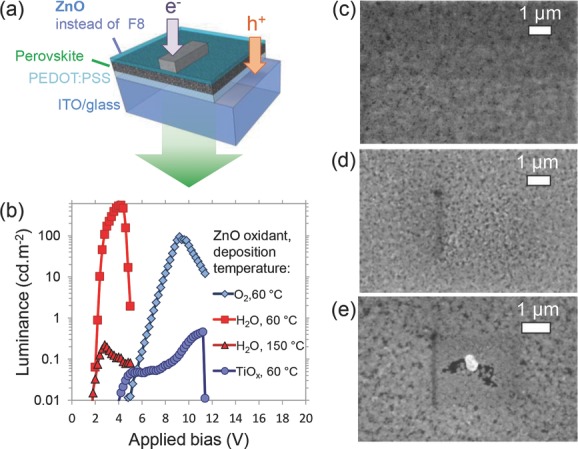
CH_3_NH_3_PbBr_3_ perovskite LEDs (PeLEDs) with ZnO or TiO*_x_* instead of F8 as the electron injector. a) Structure of PeLEDs with 20 nm Ca and 100 nm Ag top electrodes. b) Luminance versus applied bias of CH_3_NH_3_PbBr_3_ PeLEDs with SAALD ZnO deposited on top at 60 °C with O_2_ oxidant, 60 °C with H_2_O vapor oxidant and 150 °C using H_2_O vapor oxidant. PeLEDs with sol-gel TiO*_x_* electrodes are also shown. SEM images of: c) as-spun perovskite on PEDOT:PSS compared with the perovskite scanned with water vapor for 3 min at: d) 60 °C and e) 150 °C.

Depositing ZnO onto organometal halide perovskites using an ALD-based technique is typically challenging because conventional ALD requires pumping down to vacuum, which increases the deposition time and reduces scalability.[21] In particular, this would require heating the perovskite (typically between 70 °C and 150 °C)[22,23] for 30 min or more in the ALD chamber, which can detrimentally affect the substrate coverage and morphology.[24] Using SAALD allowed us to overcome these difficulties, because the metal oxide can be rapidly deposited in open air at low temperatures (150 °C or below).[22,25] We were therefore able to directly load and unload the samples from the substrate holder, resulting in the samples being heated only for the time required to deposit the films (3 min herein). This minimizes the adverse effects of heating the perovskite during deposition.

The use of water vapor as an SAALD precursor, however, presents a major drawback as organometal halide perovskites are known to react with water.[12] As a result, the reliability of the PeLEDs with SAALD ZnO deposited using water vapor as the oxidant was low, with only 15% of the devices turning on. We improved this reliability to more than 60% turning on by instead using oxygen gas as the oxidant, since perovskites are known to be stable to oxygen exposure.[26]

As is shown in 1Figure b, SAALD ZnO deposited with oxygen gas led to PeLEDs with a higher turn-on voltage of 5 V. This was primarily due to the ZnO deposited with oxygen gas having a larger electron affinity than that deposited with water vapor. This leads to the former having a smaller band gap, as measured by photoluminescence spectroscopy (Figure S3, Supporting Information), which would lead to a larger electron injection barrier with the CH_3_NH_3_PbBr_3_. In addition, ZnO deposited with oxygen gas has a higher resistivity (Table S1, Supporting Information). However, a significant benefit of producing the ZnO by SAALD is that the electron affinity can be finely tuned through Mg incorporation.[25] The comparison of energy levels presented in **Figure 2**a,b suggests that incorporating 44 at% Mg into ZnO leads to barrierless electron injection. Figure 2c shows that replacing SAALD ZnO with SAALD Zn_0.56_Mg_0.44_O reduces the turn-on voltage by approximately 1 V, to a level comparable with PeLEDs using F8.

**Figure 2:**
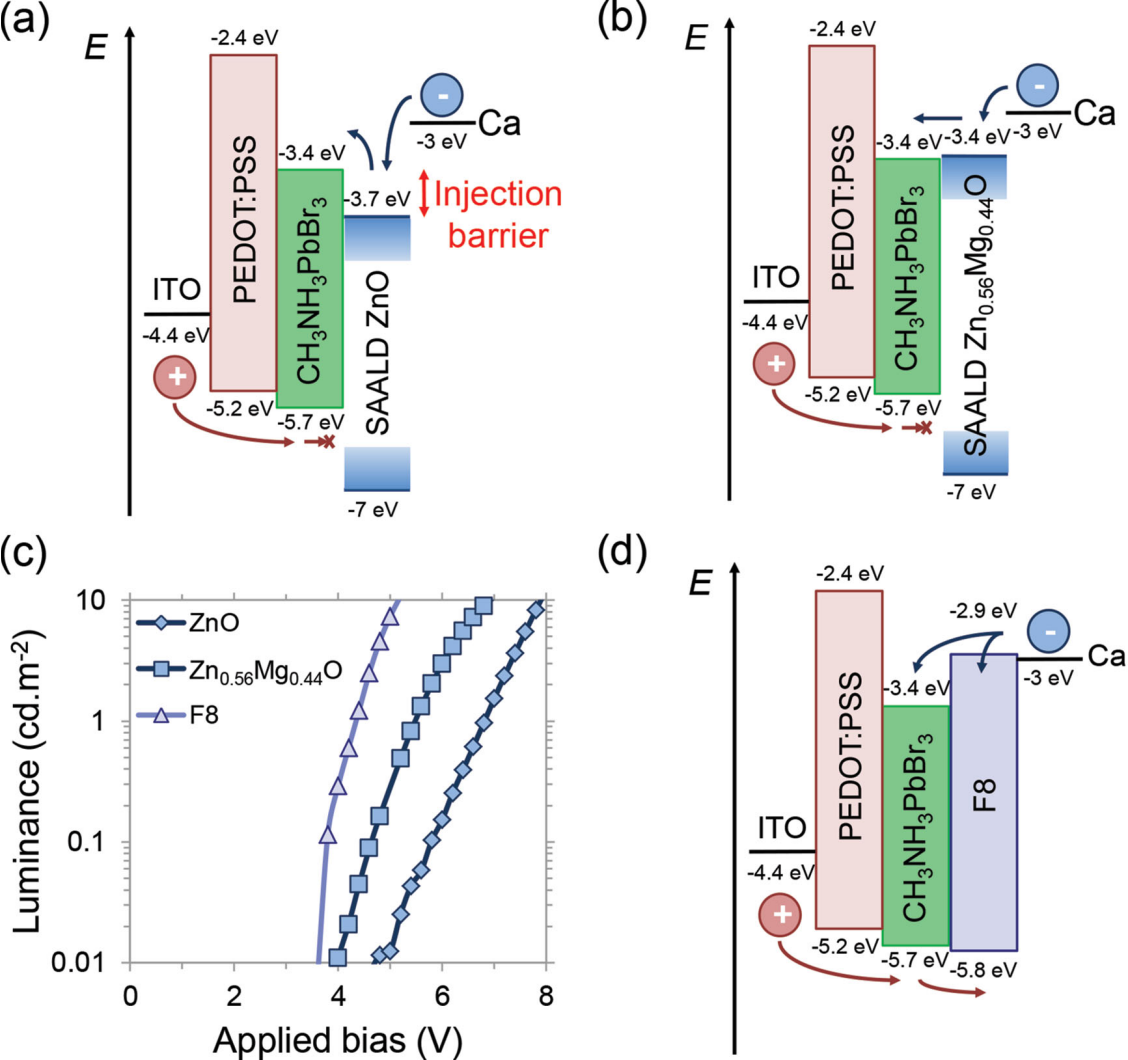
Effect of the electron-injecting material on the turn-on voltage of CH_3_NH_3_PbBr_3_ PeLEDs. a) Band diagram of PeLEDs with SAALD ZnO, showing how it forms an electron injection barrier with CH_3_NH_3_PbBr_3_ but blocks holes from the perovskite. b) Band diagram of PeLEDs with SAALD Zn_0.56_Mg_0.44_O, showing how its electron affinity is at the same level as the perovskite, giving no electron injection barrier. The deep valence band position of SAALD Zn_0.56_Mg_0.44_O also leads to the blocking of holes from the perovskite. c) Luminance versus applied bias of PeLEDs with SAALD ZnO, SAALD Zn_0.56_Mg_0.44_O, and F8. d) Band diagram of PeLEDs with F8, showing how its shallow LUMO leads to barrierless electron injection into the perovskite. However, the shallow HOMO level of F8 leads to ineffective hole blocking, allowing excitons to form and radiatively recombine in the F8. The band positions shown in this Figure are from reports in the literature.[4,25,33,34]

To investigate the advantages of replacing the polyfluorene electron injector with our tunable metal oxide, we synthesized PeLEDs with F8 using the previously reported structure (2Figure d),[4] which reproduced the previously reported performance (Figure S4, Supporting Information). The electroluminescence spectrum of these PeLEDs (**Figure 3**a) exhibited peaks at 428, 452, and 500 nm, in addition to the perovskite emission peak at 525 nm. These are due to parasitic emission from the F8, as can be seen by comparing with the normalized electroluminescence spectrum of F8-only devices in Figure 3b, and is also indicated by its band diagram in Figure 2d. In addition, when the F8 PLED was held at 9 V bias, the initially blue electroluminescence spectrum became dominated by new green peaks (at wavelengths of 520, 570, and 620 nm) after only 7 s. This green emission was irreversible, and is consistent with the formation of on-chain keto defects in the F8 due to reaction with oxygen.[14]

**Figure 3:**
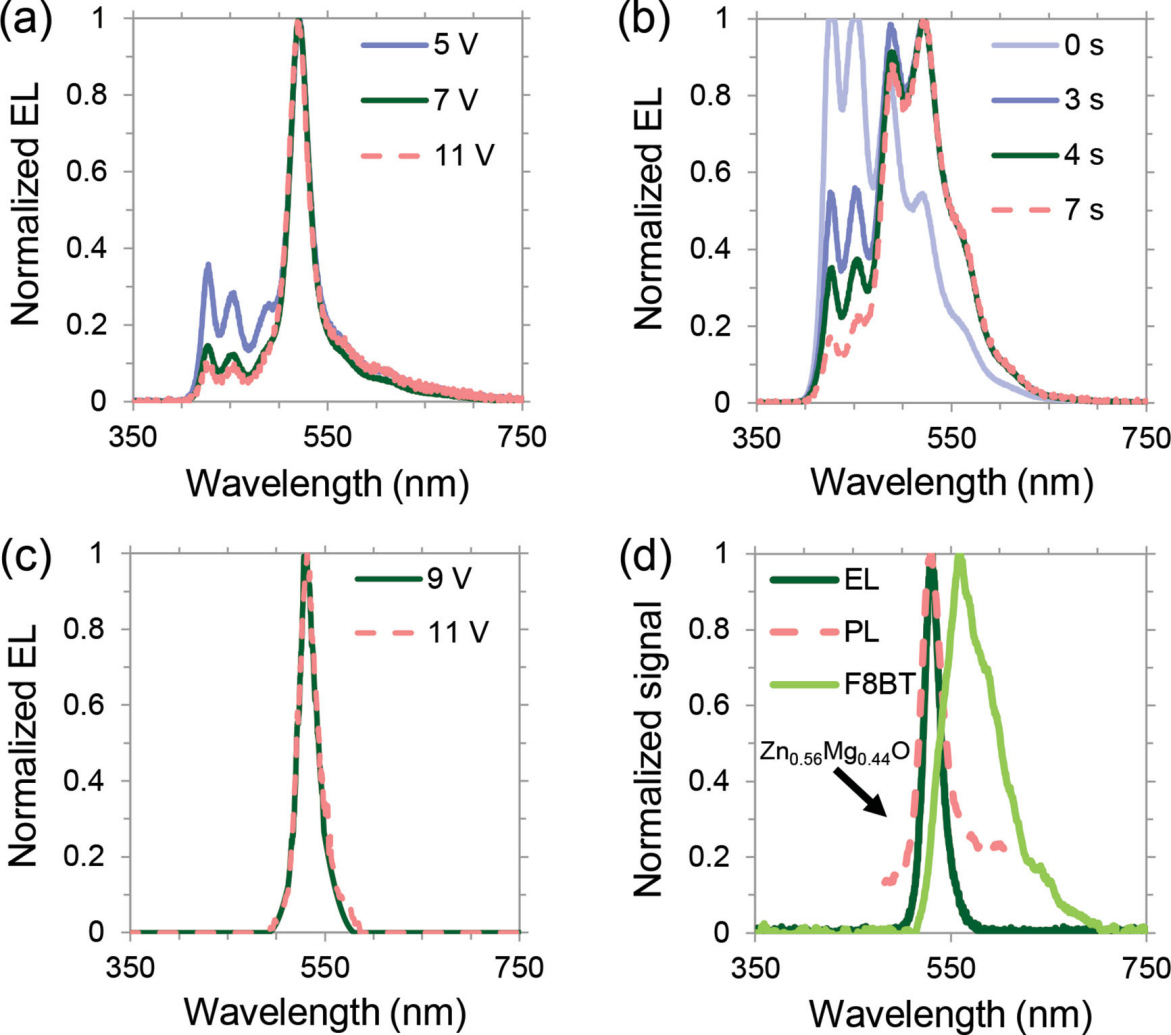
Effect of the electron injecting material on the color purity of CH_3_NH_3_PbBr_3_ PeLEDs. a) Normalized electroluminescence spectra of ITO/PEDOT:PSS/CH_3_NH_3_PbBr_3_/F8/Ca/Ag driven at 5, 7, and 11 V. b) Normalized time-resolved electroluminescence spectra of ITO/PEDOT:PSS/F8/Ca/Ag driven at 9 V after 0, 3, 4, and 7 s. c) Normalized PeLED electroluminescence spectrum with Zn_0.56_Mg_0.44_O electron injector driven at 9 and 11 V. d) Normalized electroluminescence spectrum of PeLED with Zn_0.56_Mg_0.44_O electron injector driven at 9 V compared with the normalized photoluminescence spectrum from the bare perovskite and normalized electroluminescence spectrum from an F8BT polymer LED.

Using SAALD Zn_0.56_Mg_0.44_O instead of F8 in these PeLEDs resulted in a single, well-defined electroluminescence peak at 525 nm (Figure 3c), which matched the photoluminescence peak of the bare perovskite (Figure 3d). Identical behavior was observed using SAALD ZnO deposited using either oxygen gas or water vapor as the oxidant (Figure S5, Supporting Information). We recorded the emission spectra of the PeLEDs with SAALD Zn_1−*x*_Mg*_x_*O at different applied biases (Figure 3c and Figure S5, Supporting Information) and found no change in the normalized spectra, indicating that the SAALD Zn_1−*x*_Mg*_x_*O does not degrade under bias, unlike F8.

The full width half maximum (FWHM) of the electroluminescence peak was 25 nm, which is half of the FWHM from industry standard green InGaN LEDs[27] and less than half the FWHM of the peak from a “fruit-fly” F8BT PLED (Figure 3d). The PeLED spectral emission is also sharper than that reported for some green colloidal quantum dot LEDs (>30 nm FWHM),[28] for which high color purity is considered one of their greatest strengths.[29] Zn_1−*x*_Mg*_x_*O also exhibits a deep valence band at −7 eV,[25] presenting a barrier of 1.3 eV to injected holes and thereby providing strong leakage suppression. Using SAALD Zn_1−*x*_Mg*_x_*O therefore allows PeLEDs to fulfill their potential as stable, color-pure devices with sharp emission spectra, a possibility suggested by the sharp band edges of perovskite semiconductors.[11] This is crucial for ultrahigh-definition display applications, in which a narrower spectral width of the three primary colors results in better color rendering and range.[29] Our metal oxide electron injector could also be used to achieve electrically pumped lasing from perovskites,[10] as the oxide enables electrically induced emission to occur from the perovskite only. In addition, cryogenic measurements can be performed on PeLEDs with Zn_1−*x*_Mg*_x_*O, since the metal oxides possess high carrier mobilities even at low temperatures.[30,31]

The use of organometal halide perovskites in LEDs is currently in its infancy but, if their development in the solar cells field can be taken as an example, these materials promise a wealth of discoveries that will lead to rapid efficiency developments. To demonstrate that our tunable metal oxide is high quality and could be used in efficient devices, we applied our SAALD Zn_1−*x*_Mg*_x_*O to mature PLED technology. We produced green PLEDs using emissive layers of poly(9,9-dioctyl fluorene-*alt*-benzothiadiazole) or F8BT, as well as blue PLEDs using aryl polyfluorene mixed with poly(9,9-dioctylfluorene-*co*-*N*-(4-butylphenyl)-diphenylamine) or aryl-F8:0.5 wt%TFB. Using a 60 nm SAALD Zn_0.85_Mg_0.15_O electron injector, a luminous efficiency of 21 cd A^−1^ was obtained from the green F8BT PLEDs (**Figure 4**a), which is comparable with the highest reported in the literature,[3] while a luminous efficiency of 6.8 cd A^−1^ was obtained from blue aryl-F8:0.5 wt%TFB PLEDs (Figure 4b), which is higher than the previous report of 5.9 cd A^−1^.[18] Carefully increasing the Mg content in Zn_1−*x*_Mg*_x_*O also reduced the bias required to produce 200 cd m^−2^ luminance from both types of PLEDs, while maintaining luminous efficiency (Figure 4c and Figure S6 and S7, Supporting Information). We also note that the bias producing 10 mA cm^−2^ from the blue PLEDs was reduced from 16 (using SAALD ZnO) to 9.3 V (using SAALD Zn_0.56_Mg_0.44_O), as shown by Figure 4d, which is significantly lower than the previously reported 16.9 V for aryl-F8:0.5 wt%TFB PLEDs using spray-pyrolyzed ZnO.[18] This emphasizes that our tunable metal oxide is just as effective an electron injector as spray-pyrolyzed ZnO (previously the favored cathode material for efficient hybrid LEDs),[3,18,32] but with the additional benefits of exhibiting a tunable electron injection level and the capability to be deposited at low temperature, directly onto perovskite emitters to produce PeLEDs that do not rely on fluorene-based organic transport layers.

**Figure 4:**
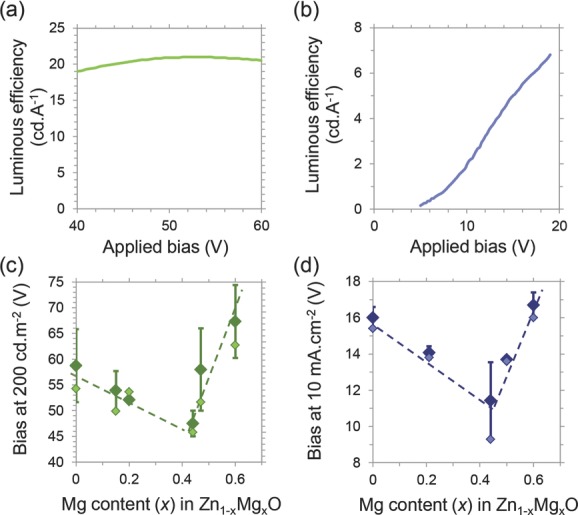
Demonstration of the potential of fluorene-free PeLED technology using SAALD Zn_1−*x*_Mg*_x_*O by applying SAALD Zn_1−*x*_Mg*_x_*O to efficient PLEDs. Luminous efficiency versus applied bias of the highest performing a) F8BT PLED and b) aryl-F8:0.5 wt%TFB PLED. Change in operating voltage with Mg content in Zn_1−*x*_Mg*_x_*O of c) F8BT and d) aryl-F8:0.5 wt%TFB PLEDs. The average (dark markers) and lowest (light markers) operating voltages measured are shown. These voltages decrease until *x* > 0.44 in SAALD Zn_1−*x*_Mg*_x_*O, when an insulating phase appears.[25]

In conclusion, we produced fluorene-free PeLEDs by adopting a very careful synthesis approach that enabled the deposition of SAALD ZnO directly onto CH_3_NH_3_PbBr_3_ perovskite. This involved using a short deposition time and low processing temperature of 60 °C, producing green PeLEDs with an improved turn-on voltage and higher luminance than those previously reported using F8. The advantages of using SAALD ZnO as the electron injector instead of F8 are: i) The ability to tune the electron injection level through Mg incorporation, reducing the turn-on voltage, ii) sharp, color-pure emission from the PeLEDs due to a larger hole injection barrier, and iii) enhanced stability in air and compatibility with cryogenic measurements. The improvements obtained with SAALD Zn_1−*x*_Mg*_x_*O make these PeLEDs highly appealing for ultrahigh definition display applications, and paves the way toward electrically pumped lasers. Being able to deposit a metal oxide with a tunable electron affinity onto perovskites could also be highly desirable for electron transport layers (ETLs) in inverted perovskite solar cells that are not reliant on fluorene-based layers,[20,24] since this enables the ETL electron energy level alignment with the absorber LUMO to be optimized to minimize energy losses.[25] SAALD Zn_1−*x*_Mg*_x_*O can play an important role in fluorene-free PeLEDs as this new field develops and their efficiencies increase to a commercially appealing level, as seen from the highly efficient green F8BT and blue aryl-F8:0.5 wt% TFB PLEDs that were produced using our metal oxide electron injector. This work therefore demonstrates a successful tunable oxide electron injector that can enable high performance in perovskite LEDs that are not dependent on expensive and less stable fluorene layers.

## Experimental Section

*Perovskite Light-Emitting Diode Fabrication*: ITO/glass (Colorado Concepts LLC) substrates were ultrasonically cleaned in acetone and isopropyl alcohol for 15 min, followed by oxygen plasma cleaning for 10 min with a Diener Low Pressure Plasma System. PEDOT:PSS (Clevios) was spin cast at 6000 rpm for 30 s and annealed at 140 °C for 30 min in a nitrogen-filled glovebox. CH_3_NH_3_PbBr_3_ (prepared according to previous reports)[4] was spin cast on the PEDOT:PSS at 3000 rpm for 30 s and annealed at 80 °C for 15 min, followed by 100 °C for 2 min. For conventional devices, F8 (Cambridge Display Technology) was spin cast from solution (10 mg mL^−1^ in chlorobenzene) on the perovskite at 3000 rpm for 30 s. For PeLEDs with SAALD Zn_1−*x*_Mg*_x_*O, the metal oxides were deposited in open air onto the perovskite at 60 °C for 3 min using either: i) nitrogen gas bubbled through deionized water at 100 mL min^−1^ and diluted with 200 mL min^−1^ nitrogen gas or ii) oxygen gas flowing at 100 mL min^−1^ to the gas manifold as the oxidant. For SAALD ZnO, nitrogen gas was bubbled through diethylzinc at 25 mL min^−1^ and diluted with 100 mL min^−1^ carrier nitrogen gas before being fed to the gas manifold. Nitrogen gas flowing at 500 mL min^−1^ was also fed to the gas manifold to form the inert gas channels. For Zn_1−*x*_Mg*_x_*O, the only difference was that the nitrogen gas was bubbled through the diethylzinc at 4 and 200 mL min^−1^ through bis(ethylcyclopentadienyl)magnesium heated to 55 °C. Sol-gel TiO*_x_* was prepared and deposited using a previously reported method.[20] To complete the devices, 20 nm Ca and 100 nm Ag were thermally evaporated through a shadow mask to give devices with an area of 5.25 mm^2^.

*Polymer Light-Emitting Diode Fabrication*: ITO/glass (Colorado Concepts LLC) substrates were ultrasonically cleaned in toluene and isopropyl alcohol for 15 min. SAALD Zn_1−*x*_Mg*_x_*O was deposited at 150 °C onto the ITO according to previous reports.[25] These films were annealed at 400 °C for 15 min. Cs_2_CO_3_ (5 mg mL^−1^ in 2-methoxymethanol) was spin cast on the annealed SAALD Zn_1−*x*_Mg*_x_*O at 6000 rpm for 45 s. For efficient F8BT PLEDs, 1200 nm F8BT (45 mg mL^−1^ in p-xylene) was spin cast on top at 2000 rpm for 45 s before being annealed at 160 °C for 1 h in a nitrogen-filled glovebox. TFB (20 mg mL^−1^ in p-xylene) was spin cast on top of both types of films at 700 rpm for 45 s. For blue PLEDs, aryl-F8 (30 mg mL^−2^ in p-xylene) had 0.5 wt% TFB mixed into it, and this mixture was spin cast at 2000 rpm for 45 s to give a 450 nm thick film, which was annealed at 120 °C for 1 h in a nitrogen-filled glovebox. For the F8BT and aryl-F8:0.5 wt% TFB PLEDs, MoO_3_ (10 nm), and Au (50 nm) were thermally evaporated on top through a shadow mask to produce 5.25 mm^2^ pixels.

*Device Characterization*: The luminous efficiencies, external quantum efficiencies, and current densities were measured using a silicon photodiode and Keithley 2400 source measure unit according to previous reports.[4] The electroluminescence spectra were also measured according to previous reports using a Labsphere CDS-610 spectrometer.[4]

*Film Characterization*: The photoluminescence of the perovskite film on ITO/PEDOT:PSS was measured with an InGaAs detector (Andor iDus DU490A). These measurements were performed in an integrating sphere under a constant stream of nitrogen and the excitation laser had a wavelength of 405 nm (Omnicron LDM405.100.CWA.L) with a power of 100 mW. Scanning electron microscopy (SEM) images of the perovskite films were obtained using a LEO VP-1530 field emission scanning electron microscope.
