# Supplementary material for: Enhanced Performance in Fluorene-Free Organometal Halide Perovskite Light-Emitting Diodes using Tunable, Low Electron Affinity Oxide Electron Injectors
Source: Adv Mater. 2015 Jan 9;27(8):1414–9. doi: 10.1002/adma.201405044 (PMC4515082; doi:10.1002/adma.201405044)
Supplement: Supplementary file 1 [file adma0027-1414-sd1.pdf]

Copyright WILEY-VCH Verlag GmbH & Co. KGaA, 69469 Weinheim, Germany, 2014.

ADVANCED MATERIALS

Supporting Information

for *Adv. Mater.*, DOI: 10.1002/adma.201405044

Enhanced Performance in Fluorene-Free Organometal Halide Perovskite Light-Emitting Diodes using Tunable, Low Electron Affinity Oxide Electron Injectors

*Robert L. Z. Hoye, Matthew R. Chua, Kevin P. Musselman, Guangru Li, May-Ling Lai, Zhi-Kuang Tan, Neil C. Greenham, Judith L. MacManus-Driscoll, Richard H. Friend, and Dan Credgington**

Copyright WILEY-VCH Verlag GmbH & Co. KGaA, 69469 Weinheim, Germany, 2014.

Supporting Information for *Adv. Mater.*, DOI: 10.1002/adma.201405044.

Enhanced Performance in Fluorene-Free Organometal Halide Perovskite Light Emitting Diodes using Tunable, Low Electron-Affinity Oxide Electron-Injectors

*Robert L.Z. Hoye, Matthew R. Chua, Kevin P. Musselman, Guangru Li, May-Ling Lai, Zhi-Kuang Tan, Neil C. Greenham, Judith L. MacManus-Driscoll, Richard H. Friend and Dan Credgington**

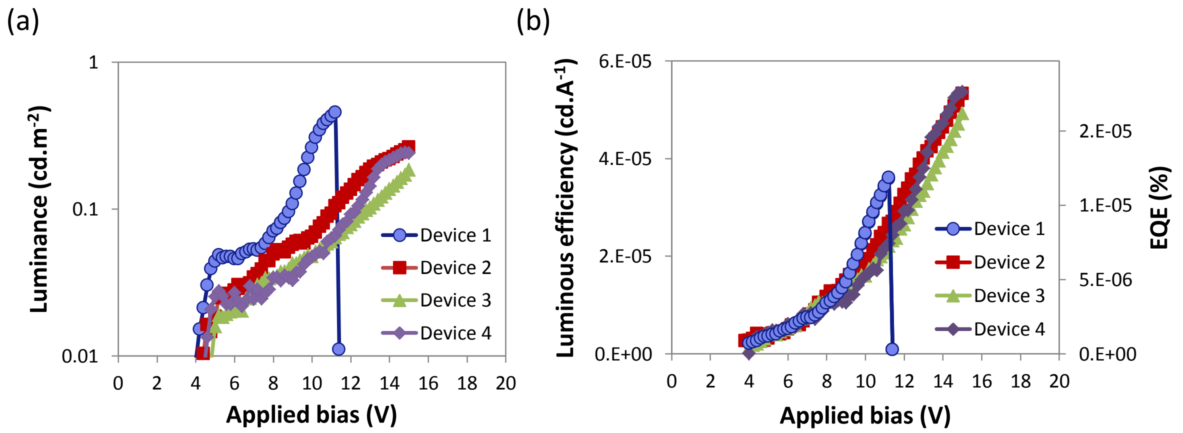

Figure S1. (a) Luminance and (b) luminous efficiency and external quantum efficiency (EQE) of ITO/PEDOT:PSS/$CH_3NH_3PbBr_3$/TiO_x/Ca/Ag perovskite LEDs (PeLEDs). The performances of the four best working devices produced under identical conditions are shown. The luminance of Device 1 is shown in Figure 1b of the manuscript. The TiO_x was spin-cast using a similar sol-gel process to that which was previously used for perovskite solar cells.[1] The same precursor solution and spin speed were used, but the films were annealed at only 60 °C for 3 min (instead of 130 °C for 10 min used in Ref. [1]) in air. We found that when the TiO_x was annealed at 130 °C for 10 min, all PeLEDs shorted, which is likely to be mainly due to the increased porosity of the perovskite under heating, as we showed in Figure 1 of the manuscript. Annealing at 60 °C for 3 min is comparable to the heating conditions we used to deposit SAALD ZnO and $Zn_{0.56}Mg_{0.44}O$. However, it can be seen that the luminance of all devices with TiO_x were below 1 cd.m^{-2} and therefore at least 2 orders of magnitude lower than the luminance of our PeLEDs with SAALD ZnO produced under optimum conditions (Figure 1b of the manuscript). The luminous efficiencies and EQEs of the PeLEDs with TiO_x were also 4 orders of magnitude lower than our PeLEDs with SAALD $Zn_{1-x}Mg_xO$. The significantly lower performance of the PeLEDs with sol-gel TiO_x was due to the TiO_x precursor solvents damaging the perovskites, as shown in Figure S2.

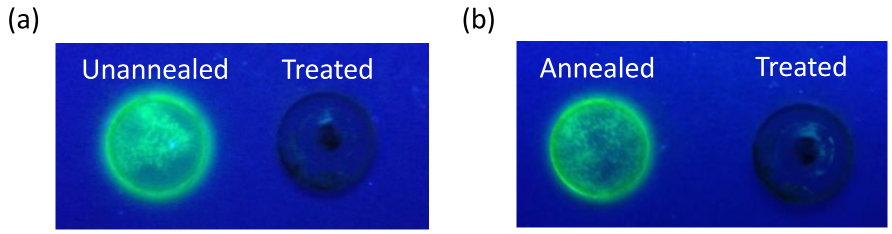

Figure S2. Photographs showing green photoluminescence (or a lack of) from $CH_3NH_3PbBr_3$ films prepared on quartz in the same way as for the perovskite used in PeLEDs with SAALD $Zn_{1-x}Mg_xO$. A comparison is drawn between bare $CH_3NH_3PbBr_3$ perovskite and treated perovskite. The treated perovskite had isopropanol with 0.013 mol.L^{-1} $HCl_{(aq)}$ spin-cast on top at 3000 rpm for 45 s (i.e.: the same solvents and spin-casting conditions for depositing sol-gel TiO_x,[1] but without any titanium isopropoxide added), followed by annealing in air at 60 °C for 3 min. We observed by eye that the perovskite films remained on the substrate after treatment with the solvents. The comparisons are between (a) as-prepared (unannealed) perovskite with the treated perovskite, and (b) perovskite annealed at 60 °C for 3 min in air with the treated perovskite. These show that treating the perovskite with aqueous HCl diluted in isopropanol causes degradation and almost no photoluminescence to occur when irradiated with UV-light (365 nm wavelength), which may explain why PeLEDs with sol-gel TiO_x had low performance. By contrast, the as-deposited and annealed perovskites without treatment displayed photoluminescence under UV-light.

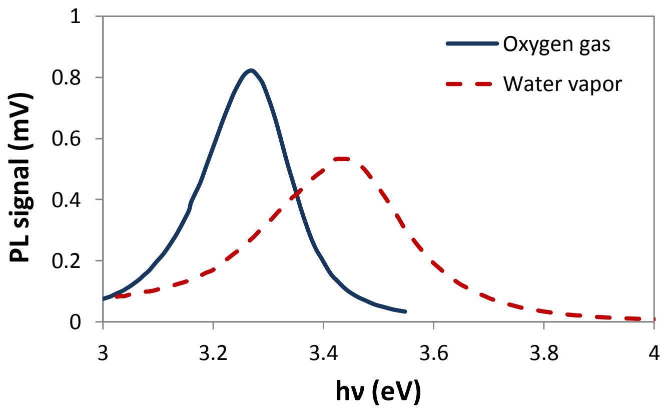

Figure S3. Photoluminescence (PL) measurements of SAALD ZnO deposited at 60 °C using oxygen gas and water vapor as the oxidant. These were obtained using an ACCENT RPM 2000 system with a Nd:YAG laser (266 nm wavelength, 4.5 mW power).

Table S1. Out-of-plane resistivity of SAALD ZnO and $Zn_{0.56}Mg_{0.44}O$ deposited at 60 °C and 150 °C using O_2 compared with H_2O vapor as the oxidant. These resistivities were obtained by performing *I – V* sweeps on the films. ITO was used as the back contact and 20 nm Ca with 100 nm Ag as the top contact. The resistance of ITO/Ca/Ag was subtracted.

Material	Deposition temperature (°C)	Oxidant	Resistivity (kΩ.cm)
ZnO	60	O_2	28 $\pm$ 8
$Zn_{0.56}Mg_{0.44}O$	60	O_2	90 $\pm$ 30
ZnO	150	O_2	13 $\pm$ 5
$Zn_{0.56}Mg_{0.44}O$	150	O_2	12 $\pm$ 3
ZnO	60	H_2O	10 $\pm$ 3
$Zn_{0.56}Mg_{0.44}O$	60	H_2O	49 $\pm$ 15
ZnO	150	H_2O	7 $\pm$ 1
$Zn_{0.56}Mg_{0.44}O$	150	H_2O	9 $\pm$ 3

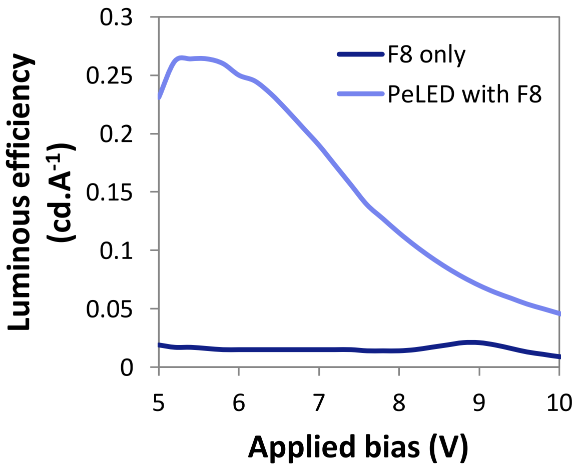

Figure S4. Luminous efficiency vs. applied bias of ITO/PEDOT:PSS/$CH_3NH_3PbBr_3$/F8/Ca/Ag devices compared with ITO/PEDOT:PSS/F8/Ca/Ag devices. The luminous efficiency of the F8 PLEDs were an order of magnitude lower than that of the PeLEDs, indicating that the PeLED emission was dominated by that from the perovskite. But for quality display applications (and ultimately electrically-pumped lasing), it is essential to remove the contamination of the perovskite electroluminescence by the F8 emission. The $CH_3NH_3PbBr_3$ here was annealed at 100 °C for 15 min in a nitrogen-filled glovebox.

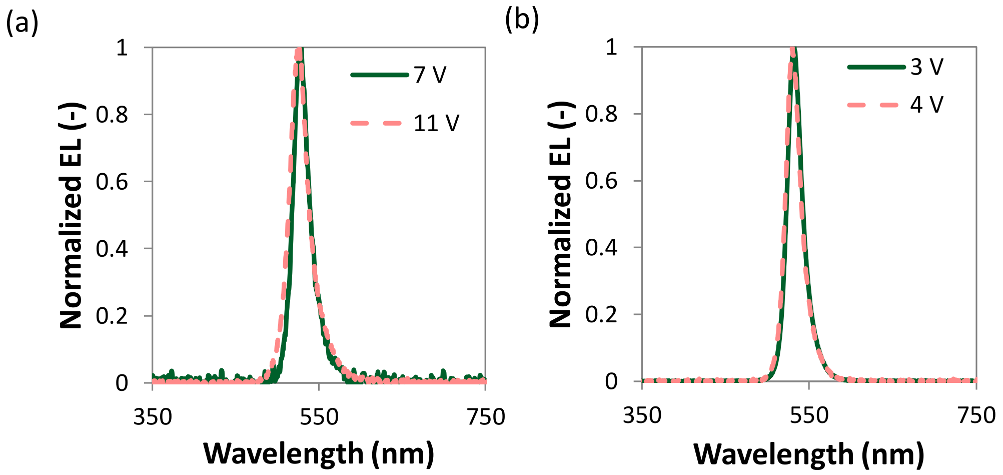

Figure S5. (a) Normalized electroluminescence spectrum of a $CH_3NH_3PbBr_3$ PeLED with SAALD ZnO deposited using oxygen gas as the oxidant biased at 7 V and 11 V. The CIE co-ordinates of this device were (0.17, 0.74). (b) Normalized electroluminescence spectrum of $CH_3NH_3PbBr_3$ PeLED with SAALD ZnO deposited using water vapor as the oxidant and biased at 3 V and 4 V. Smaller biases were applied because the turn-on voltage (2 V) was lower.

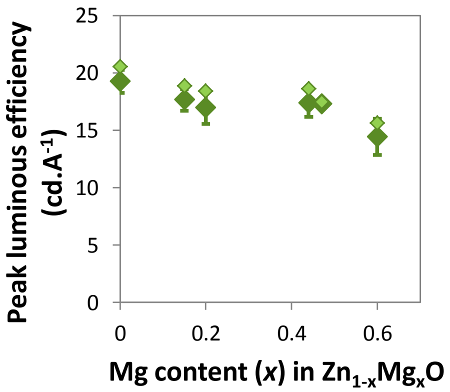

Figure S6. Change in the peak luminous efficiency of PLEDs with 1000 nm F8BT with the Mg content of the SAALD $Zn_{1-x}Mg_xO$ electron-injector. The efficiency is reduced for Mg contents above 44 at.% because an insulating rocksalt phase forms.[2]

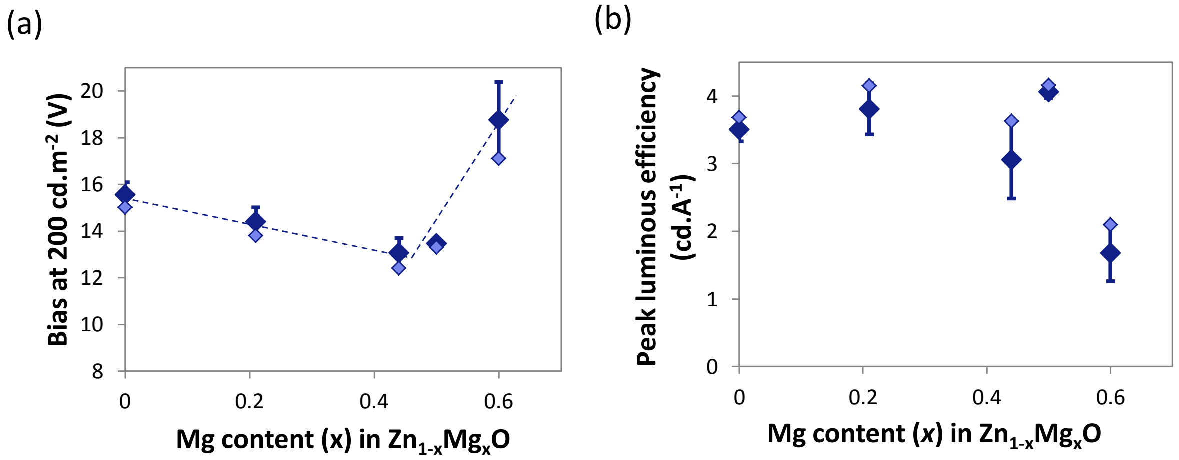

Figure S7. (a) Change in the applied bias required to produce 200 cd.m^{-2} and (b) peak luminous efficiency from aryl-F8:0.5wt.%TFB PLEDs with Mg content in SAALD $Zn_{1-x}Mg_xO$ electron-injectors. The applied bias increases and peak luminous efficiency decreases for $x > 0.44$ in SAALD $Zn_{1-x}Mg_xO$ due to the appearance of the insulating rocksalt phase.[2]

References

[1] P. Docampo, J. M. Ball, M. Darwich, G. E. Eperon, H. J. Snaith, *Nat. Commun.* **2013**, *4*, 2761.

[2] R. L. Z. Hoye, B. Ehrler, M. L. Böhm, D. Muñoz-Rojas, R. M. Altamimi, A. Y. Alyamani, Y. Vaynzof, A. Sadhanala, G. Ercolano, N. C. Greenham, R. H. Friend, J. L. MacManus-Driscoll, K. P. Musselman, *Adv. Energy Mater.* **2014**, *4*, 1301544.
